# Supplementary material for: mmannot: How to improve small–RNA annotation?
Source: PLoS One. 2020 May 28;15(5):e0231738. doi: 10.1371/journal.pone.0231738 (PMC7255610; doi:10.1371/journal.pone.0231738)
Supplement: S1 File — (PDF) [file pone.0231738.s001.pdf]

Versions of the tools

- bedtools 2.27.1
- bowtie: 1.2.2
- bwa: 0.7.17-r1188
- FASTX Toolkit: 0.0.14
- Python: 2.7.16
- R: 3.6.1
- samtools: 1.9
- ShortStack: 3.8.5

Releases of the genome assemblies

- *Arabidopsis thaliana*: TAIR10
- *Danio rerio*: GRCz10
- *Homo sapiens*: GRCh38
- *Sus scrofa*: 11.1

Scripts

Trimming and cleaning

fastq2fasta

```
#!/usr/bin/env python
import fileinput

for cpt, line in enumerate(fileinput.input()):
	line = line.strip()
	if cpt % 4 == 1:
		seq = line
	elif cpt % 4 == 3:
		print ">_%s\n%s" % (line, seq)
```

removeCR

```
#!/usr/bin/env python
import fileinput

s = False
for line in fileinput.input():
        line = line.strip()
        if line[0] == '>':
                if s: print s
                print line
                s = ""
        else:
                s = "%s%s" % (s, line)
print s
```

fasta2fastq

```
#!/usr/bin/env python
import fileinput

for cpt, line in enumerate(fileinput.input()):
        line = line.strip()
        if cpt % 2 == 0:
                n = line[2:]
        else:
                print "@seq_%d\n%s\n+\n%s" % (cpt/2+1, line, n)
```

Pipe-line

```
fastx_clipper -a adapter -l 15 -i reads.fastq | fastq2fasta > tmp;
dust tmp | removeCR | sed 'N;s/\n/|/g' | awk '(length($0) >= 32)' |
     grep -v N | sed 's/|/\n/g' | fasta2fastq > reads_trimmed.fastq
```

Mapping

Mapping with BWA

```
bwa aln -n 2 -N -i 1 -R 5000 -t 6 genome_index reads.fastq > reads.sai
bwa samse -n 5000 genome_index reads.sai reads.fastq* | samtools view -uS -
    | samtools sort -o \textit{reads.bam} -
```

Mapping with bowtie

```
bowtie -S -a --best --strata -m 20 -p 6 genome_index reads.fastq |
    addNH | samtools view -uS - | samtools sort -o reads.bam -
```

Quantification strategies

mmannot

```
/usr/bin/time -o results.time mmannot -c configuration_file -a
    annotation.gtf -r reads.bam -l 0.6 -p -y strategy -m
    results_stats1.csv -M results_stats2.csv > results.csv
```

The strategy can be `unique`, `ratio`, `random`, or `default`.

MMR MMR only works with the BAM files produced by bowtie.

```
samtools sort -@ 6 -n reads.bam -o reads_sorted.bam
mmr -o reads_MMR.bam -t 6 -S -b reads_sorted.bam
mmannot -c configuration_file -a annotation.gtf -r reads_MMR.bam -l 0.6 -p -y unique > results.csv
```

ShortStack

```
ShortStack --bowtie_cores 6 --nohp --align_only --readfile reads.fastq --genomefile genome_index
mmannot -c configuration_file -a annotation.gtf -r reads_ShortStack.bam -l 0.6 -p -y unique > results.csv
```

Extracting annotation of sRNAs from standard GFF/GTF files This was used for the *Arabidopsis thaliana* dataset. It has been adapted to other datasets.

```
#!/usr/bin/env Rscript

library(rtracklayer)
library(GenomicFeatures)
library(readr)
library(magrittr)
library(dplyr)

args = commandArgs(trailingOnly=TRUE)
if (length(args) != 2) {
  stop("Two parameters (input GFF/GTF) and output (BED) files are expected")
}
inputFileName <- args[1]
outputFileName <- args[2]

annotationGR <- import(inputFileName)
annotationDb <- makeTxDbFromGFF(inputFileName)
annotations <- list()
annotations[["3'UTR (+)"]] <- unlist(threeUTRsByTranscript(annotationDb))
annotations[["5'UTR (+)"]] <- unlist(fiveUTRsByTranscript(annotationDb))
annotations[["CDS (+)"]] <- cds(annotationDb)
annotations[["intron"]] <- unlist(intronsByTranscript(annotationDb))
geneAnnotation <- annotationGR[annotationGR$type == "gene"]
annotations[["upstream"]] <- flank(geneAnnotation, start = TRUE, width = 1000)
annotations[["downstream"]] <- flank(geneAnnotation, start = FALSE, width = 1000)
for (name in c("miRNA", "tRNA", "snoRNA", "snRNA", "rRNA",
```

```r
                "transposable_element_gene", "pseudogenic_transcript")) {
  annotations[[name]] <- annotationGR[annotationGR$type == name]
}
for (annotationType in names(annotations)) {
  if (!(grepl("(+)", annotationType, fixed = TRUE))) {
    tmpAnnot <- annotations[[annotationType]]
    strand(tmpAnnot) = ifelse(strand(tmpAnnot) == "+", "-", "+")
    annotations[[annotationType]] <- append(annotations[[annotationType]], tmpAnnot)
  }
}
geneAntisense <- geneAnnotation
strand(geneAntisense) <- ifelse(strand(geneAnnotation) == "+", "-", "+")
annotations[["gene (-)"]] <- geneAntisense
for (name in names(annotations)) {
  if (length(annotations[[name]]) == 0) {
    annotations[[name]] <- NULL
  }
  else {
    mcols(annotations[[name]]) <- NULL
    annotations[[name]]$type <- name
  }
}
mergedAnnotations <- unlist(as(annotations, "GRangesList"))
data.frame(name   = mergedAnnotations$type,
           chr    = seqnames(mergedAnnotations),
           start  = start(mergedAnnotations),
           end    = end(mergedAnnotations),
           strand = strand(mergedAnnotations)) %>%
  filter(start > 0) %>%
  distinct() %>%
  write_tsv(path = outputFileName,
            quote_escape = FALSE,
            col_names = FALSE)
```

FeatureCounts

```
featureCounts -a annotation.saf -F SAF reads.bam --fracOverlap 0.6 -o output.fc -L -s 1
```

Generating a database of sequences of sRNAs and the genome

```
# SAF to BED conversion
awk -F '\t' '{print($2 "\t" ($3-1) "\t" $4 "\t" $1 "\t.\t" $5)}' annotation.saf | sort -u > annotation.bed
# Generating the database of sequences of sRNAs
bedtools getfasta -fi genome.fasta -bed annotation.bed -name -s > annotation.fasta
# Adding the genome
sed 's/>.*/>genome/g' genome.fasta > tmp.fa
cat annotation.fasta tmp.fa > tmp2.fa
mv -f tmp2.fa annotation.fasta
```
