# Supplementary material for: mmannot: How to improve small–RNA annotation?
Source: PLoS One. 2020 May 28;15(5):e0231738. doi: 10.1371/journal.pone.0231738 (PMC7255610; doi:10.1371/journal.pone.0231738)
Supplement: Attachment — Submitted filename: response.pdf [file pone.0231738.s006.pdf]

Dear Reviewer,

First, we would like to thank you for providing us crucial feedback in order to improve the quality of the article.
We counted the number of reads used by our method and other methods, and we tested (using a one-sided Wilcoxon signed-rank test) if we used significantly more reads.
The p-value are always not greater than 2e-4.
These results are summarized in a new Table, which we added in the manuscript.
We hope that you will find this revision acceptable.

Best regads,

Christine Gaspin and Matthias Zytnicki.
